# Grand Challenges in Organ Transplantation

**DOI:** 10.3389/frtra.2022.897679

**Published:** 2022-05-06

**Authors:** Jerzy W. Kupiec-Weglinski

**Affiliations:** The Dumont-UCLA Transplant Center, Division of Liver and Pancreas Transplantation, Department of Surgery, David Geffen School of Medicine at UCLA, Los Angeles, CA, United States

**Keywords:** organ transplantation, organ quality, ischemia-reperfusion injury, organ preservation, xenotransplantation, chronic rejection, immunosuppression

## Introduction

The modern era of organ transplantation was pioneered at the Peter Bent Brigham Hospital in Boston by physician-scientists John P. Merrill (nephrologist), and Joseph E. Murray (surgeon). Their joint effort led to the first successful human kidney transplantation between identical twins (1954), the first allograft between fraternal twins (1959) and the first kidney allograft from a cadaveric donor (1962). Other landmarks in transplant history include the first successful human liver (1967) and pancreas (1968) transplants, the first transplantation of the heart (1967), lung (1983), intestine (1987), and more recently hand (1998), facial tissue (2005), and the uterus (2013). Hematopoietic cell transplants were implemented in 1957 using bone marrow grafting in cancer patients. After the mechanism of acute graft rejection was confirmed to be immune-mediated, the application of immunosuppressive drugs (corticosteroids, calcineurin inhibitors) and biological modalities (e.g., polyclonal and monoclonal antibodies) paved the way for transplantation to become the standard of care for patients with end-stage organ diseases. Several Nobel Prizes have been awarded to transplant “founding fathers,” Peter B. Medawar and Frank Macfarlane Burnet (1960), Baruj Benacerraf, Jean Dausset, and George D. Snell (1980), as well as Joseph E. Murray, and E. Donnall Thomas (1990). The Lasker Award was shared by Thomas E. Starzl, and Roy Calne in 2012.

Organ transplantation remains one of the most spectacular and consequential fields in 21st-century medicine, integrating advances in surgery, immunology, genetics, pharmacology, intensive care medicine, epidemiology, and ethics. The idea of prolonging life and wellbeing through organ transplantation captures worldwide attention of medical practitioners, students, and scientists alike. Today, organ transplantations are a common feature of medical practice in developed countries and increasingly in developing countries, and new advances in the field are frequently reported in the lay press.

## Current Challenges in Organ Transplantation

Despite continued advances in our appreciation of the complexity of immune cascades and molecular cross-regulation, essential caveats and gaps in knowledge remain. In practice, these present limitations for clinical organ transplantation, but at the same time ample opportunities for insightful and ground-breaking research.

## Donor Organ Supply

The shortage of donor organs represents the most challenging global problem. Although the number of transplants during the last three decades increased 2-fold, the number of patients on waiting lists increased 6-fold. For instance, in 2021, according to the UNOS database, there were 41,354 transplants performed in the US. However, 116,566 patients remained on waiting lists while another 6,564 died waiting for the life-saving organ. Despite over 6,500 living donor transplants and 13,800 deceased donations during that period, the demand for donor organs by far exceeds the supply. Alleviation of the donor organ shortage and expansion of the transplant donor pool are important challenges that must be faced.

### Targeting Peri-Transplant Donor Tissue Damage

The major contributing factor to the organ shortage is impaired donor tissue quality due to the aging of the population and preexisting diseases (e.g., nonalcoholic steatohepatitis, NASH). These suboptimal grafts are particularly susceptible to ischemia-reperfusion injury (IRI), the innate immune-driven tissue damage during cadaver-organ harvesting (1). As many donor organs are never transplanted due to inferior quality (there were 4,994 discards in the US in 2018), there is consensus that IRI is an important contributor to the donor organ shortage. Indeed, even if successfully transplanted, these suboptimal organs experience a high incidence of primary non-function (PNF), early allograft dysfunction (EAD), and late rejections. Hence, improving donor organ quality and targeting peri-transplant tissue IRI is of paramount importance to save lives, benefit patient outcomes, and enhance the overall success of transplantation. Access to living-related donors is essential to allow borderline organs to be successfully utilized. This is particularly important for recipients that may already have received one transplant but need to be re-transplanted.

The current prevailing paradigm is to treat transplant patients with immunosuppressive agents to counter the immune rejection response. A somewhat unconventional approach would be to “rejuvenate” donor organs in the peri-transplant period and improve their quality by mitigating IRI stress, and promote tissue regeneration *via* specific molecular signaling pathways (e.g., anti-oxidant) (2). A stepwise bench-to-bedside translational approach is needed to advance the concept of “one transplant for life” by utilizing state-of-the-art biological probes (gene regulation; stem cell therapy) and bioengineering in clinically-relevant settings (3). The available arsenal of technologies (e.g., T-cell receptor sequencing, solid-phase Ab monitoring, donor-derived cell-free DNA detection), combined with the introduction of novel therapeutic targets and biomarkers, should aid in predicting clinical outcomes. It is believed that the knowledge from translational transplant studies is likely to be broadly applicable because IRI phenotypes share common mechanisms with other diseases, such as ischemic stroke and myocardial infarction. There is accumulating evidence that damage-associated molecular patterns (DAMPs) and counterbalancing suppressing DAMPs (SAMPs) initiate and control inflammation-promoting and inflammation-resolving defense responses not only in solid organ transplants but also during cancer immunotherapy (4).

### Organ Preservation

Static cold storage (SCS) in the UW solution, developed by Folkert Belzer at the University of Wisconsin some 40 years ago, remains the gold standard of organ preservation for transplantation. However, newly introduced machine perfusion techniques are fast gaining traction as an alternative (5). These are primarily relevant to marginal organs from donation after circulatory death or extended criteria donors; they extend the time of *ex-vivo* preservation, and enable objective assessment of tissue quality and viability. Hypothermic machine perfusion (HMP) safely preserves donor organs and enhances mitochondrial recovery and function, while adding oxygen in hypothermic oxygenated machine perfusion (HOPE) further improves organ preservation. By keeping the organs in a low metabolic state, while allowing the system to supply substrates and remove metabolites produced during preservation, both techniques auger well for the future as they demonstrate promise, especially for mitigating biliary complications in the DCD liver, a major complication in hepatic transplants. However, evidence is still lacking whether this approach extends donor organ preservation time, an essential consideration in the context of increasing transplant volumes and operating room logistics. Importantly, HOPE might further fulfill the remaining metabolic demand, restore tissue energy reserves and reduce oxidative stress in the peri-transplant period.

Much interest has recently been focused on normothermic machine preservation (NMP) (6). Its principle is to recreate the physiological environment by maintaining normal organ temperature, providing essential substrates for metabolism, oxygenation, and nutrition, while allowing organ recovery and functional tissue testing *ex-vivo* before transplantation. With hepatic steatosis the principal reason for a donor liver being discarded (about 1,000 livers are declined every year in the US for that reason), the NMP was identified as an effective method for reducing the fat content of high-risk livers. NMP might provide a platform where adjunctive pharmacological, gene and stem cell therapy is administered to repair suboptimal grafts. Outside the transplant field, another potential of NMP is that it could be used for elucidating mechanisms underpinning various disease pathologies and to design refined interventions.

### Revisiting Xenotransplantation to Solve Donor Organ Shortage

Except for a single chimpanzee kidney and a baboon liver, which survived 9 months and 70 days, respectively, primate-to-human kidney, heart, and liver transplants performed from the 1960s until the 1980s generally failed soon after surgery due to a size mismatch and fulminant rejection. However, significant advances in genetic engineering and immunology have rekindled xenotransplant research (7). Indeed, the advent of CRISPR/Cas9 genome editing has enabled defined pathogen-free (DPF) pigs to create multiple genetic modifications. The deletion in donor pigs of 1-3αGal and swine leukocyte antigen [SLA] combined with transgenic overexpression of specific human genes (e.g., complement regulator or thrombomodulin) helped to reduce the risk of otherwise hyperacute rejection and minimize post-transplant organ dysfunction. Minimizing the infection risk combined with refined immunosuppression, donor organ conditioning, and genome editing have markedly improved pig-to-primate transplant outcomes (e.g., 945-day survival of heterotopic pig heart in baboon recipients).

In September 2021, a team at New York University transplanted a gene-edited swine kidney into a brain-dead human recipient; the new kidney sustained blood flow and produced urine until the study concluded 77 h later. Similarly, a heart xenotransplant in a human recipient, performed in January 2022 at the University of Maryland Medical Center, used a DPF donor pig with 10 genetic modifications. Although the patient died 2 months after the surgery, this first pig-heart transplant was a landmark moment in medicine. Theoretically, organ supply from DPF pigs, with close anatomical/physiological similarities to humans, could be unlimited, procured on-demand, with minimized risks of zoonoses or human pathogen transmission, while devoid of the stresses of trauma or brain death seen otherwise in a human donation.

## Chronic Rejection in Organ Transplantation—an Undefined Conundrum

Despite advances in all aspects of organ transplantation, evidenced by the 1-year patient and graft survival often exceeding 90%, the long-term perspectives of transplantation remain problematic as the rate of decline of successfully transplanted organs has not changed in over 20 years (8). While tremendous new knowledge has been created about the cellular and molecular immune mechanisms, the field, so far, has largely failed to exploit these advances to deliver safer and more effective FDA-approved therapeutics to control transplant rejection. The standard of care (SOC) of chronically administered, FDA-approved anti-rejection agents (DNA synthesis inhibitors [AZA, MMF/MPA], CNIs [CsA, Tac), steroids) has remained the same for >25 years, and as a result, the recipient and renal transplant 5-year survivals for deceased donor transplants have improved <5% over the last 20 years (9). The accruing data from clinical trials strikes against a great hope that the new knowledge of T cell costimulation blockade would lead to improved therapeutics. This status quo highlights the need for out-of-the-box novel therapeutic strategies and the development of biomarkers for non-invasive graft function screenings. Technological advances (multiparametric flow cytometry/cell-sorting, transcriptomic, and other “-omics” signatures) remain untapped value in that quest.

As solid grafts eventually succumb to progressive vascular and interstitial inflammation/ scarring, commonly termed chronic rejection, its putative mechanism remains a fertile ground for new immune concepts to be tested in translational studies. The failure of the current “T-cell centric” paradigm prompted interest in dissecting the innate—adaptive immune interface as an alternative mechanism in chronic graft failure (10). Recent findings highlight the contribution of donor-specific antibodies (DSA) and interrelated innate signaling pathways, with a particular focus on pro-inflammatory macrophages, natural killer (NK) cells, and the complement system. The roles of an emerging immune cell family, i.e., innate lymphoid cells (ILC1-3), warrant further studies in transplant models. Surprisingly, innate immune cells acquire features of adaptive cells that either directly sense allogeneic non-self or become trained in the allogeneic milieu to promote a myriad of memory recall responses (11). Hence, adaptive features of trained myeloid cells may provide novel therapeutic opportunities not only in the transplantation field but also for treating cancers and autoimmune diseases. Future targeting of innate memory should not compromise the host protective immunity and local immune surveillance.

In addition to the innate—adaptive immune interface at the recipient site, intracellular signals and molecular events in the donor graft itself also shape the local microenvironment to promote pro-inflammatory or immunoregulatory functions. For instance, the donor brain death (BD) should no longer be considered a static condition but a dynamic process that influences donor organ quality and outcomes (12). Further definition of inflammatory mediators and acute-phase proteins in peripheral organs of BD donors is warranted to introduce donor-related therapeutics to improve the long-term graft function and survival of the recipient.

The field of nanotechnology has spurred interest, particularly in the cancer field, to improve drug pharmacokinetics, increase efficacy with minimization of systemic toxicity through therapeutic payload delivered to the tissue site, and to temporary control drug release. Indeed, proof-of-concept experiments highlight the draining lymph nodes, the primary site of alloimmunity activation, as an important target site for selective nanotherapeutics (13). This exciting first-in-class application of engineered nanoparticles to promote Treg development and possibly induce tolerance, the “holy grail” of transplantation, remains to be further developed before its translation into the clinic.

The human gastrointestinal (GI) tract microbiota, a diverse conglomerate of ca. 100 trillion bacteria (1–2 kg in mass) encompassing ca. 150-fold more genes than the human genome itself, regulates pathogen colonization, nutrient metabolism, and immunity. Indeed, modulation of the host microbiome has become an emerging strategy in transplant recipients (14). As immunosuppressive agents often induce bacterial infections caused by multidrug-resistant organisms (MDROs), the management of the gut microbiota remains a challenge. The use of bacteriophage therapy (BT), largely forgotten in the antibiotics (Abx) era, is an option for targeting MDROs (15). However, many questions remain: How does Abx treatment change the gut-microbiota composition, and what species are most affected? What molecules from the presence of beneficial, or loss of harmful, microbes influence transplant outcomes? Is there a role for pre- or post-probiotics to alter gut microbiota diversity? What organs should be the focus of microbiota-centered studies? How does altering the microbiome diversity influence immune responses in the donor organ? What are the long-term consequences of personalized bacterio (phage) therapies? Future research should also focus on the relationship between dysbiosis, altered bacterial communities, and the molecules they produce, as the knowledge from the field continues to improve the lives of those affected by organ replacement therapies.

## Conclusion

By paraphrasing the allegorical painting by a French artist, Paul Gauguin, “Where Do We Come From? What Are We? Where Are We Going?” This editorial is intended to be a snap-shot of where the things stand and what needs to be done in the transplant field. On a personal note, as a physician-scientist, I was privileged to be its witness for well over four decades, starting at the Brigham at Harvard, the original transplant mecca, and then at UCLA, today the largest organ transplant center in the country. I will take the liberty to share some thoughts based on my professional journey and experience.

First, the collaboration between basic scientists and clinicians is essential to advance the transplant field. In the new era of bioinformatics, artificial intelligence, and 3D-printed organs, looking back on my early studies with the “miracle” drug cyclosporine in the early 1980s, I am reminded that we can only benefit from mutually beneficial crosstalk and cooperative support between “Big Pharma,” the biotech industry, and academia. The unbridled energy and fresh perspectives of students, the drive of the biotech industry and the experience of “Big Pharma,” are all essential ingredients for the discovery and advance process. Our field is not unique in this respect, but the idea of prolonging life through organ transplantation is unique and continues to drive our motivation. Instead of working in silos, the fields of autoimmunity, transplant, tumor, and infectious immunity need to coordinate research and care in these disorders.

Second, major milestones from the past, sometimes long-forgotten, provide a fertile ground for new discoveries. Without the lymphocyte recirculation studies by James Gowans in the 1960s, nano-targeted drug delivery and immunomodulation *via* high endothelial venules (HEV) of draining lymph nodes in transplant recipients, as discussed before, would have been impossible to accomplish five decades later. In the late 1980s, together with Terry Strom at Harvard and Tibor Diamantstein at the West Berlin Free University, we introduced the concept of IL-2 receptor (IL-2R) targeted therapy in organ transplantation. Unaware that anti-IL-2R (p55 chain) Abs target Treg (or T suppressor cells referred to at that time), we were puzzled by the not-so-robust therapeutic effects in renal transplant trials reported by Jean-Paul Soulillou in Nantes. The subsequent progress in molecular immunology, which taught us what we were missing, ultimately led to the inclusion of anti-CD25 mAb in the clinical immunosuppressive armamentarium a decade later (Basiliximab, Daclizumab).

Third, the field of solid organ transplantation has progressed from 1-year graft survival of <40% to over 90%, and from the thoracic duct drainage/total body x-irradiation to nano-technology based anti-rejection therapies. The bioengineered pig organs seem to be a viable option to “cheat mother nature” to treat human end-stage diseases successfully, and proving those claiming “xenotransplants have great future and always will” were mistaken (including myself). Although these could dramatically improve or even solve the current organ shortage and decrease waitlist mortality, animal-to-human transplants as a treatment of choice, or a bridge to allotransplantation, raise a myriad of immunological, ethical and regulatory issues (16). The problem should be approached on several fronts: encouraging more humans to become organ donors should proceed in parallel with educating the lay audience to de-stigmatize the use of non-human organs along with the development of improved bioengineered pigs.

Fourth, despite early skepticism, limbs, faces, penises, and uteri are being transplanted clinically, pushing the boundaries of science, and initiating previously unheard dialogue in the general community. However, some unexpected and without precedence problems emerge. The first hand transplant recipient feeling “mentally detached” from it, demanded the new hand be amputated 3 years after the successful surgery by Max Dubernard in Lyon. The first, also Dubernard's, face transplant patient died 11 years later, battling mood swings and bouts of cancer linked to immunosuppression. Do the functional benefits of non-life-saving vascularized composite allografts (VCA) justify their risks related to immunosuppression and physical identity changes? Despite a restricted number of surgeries performed to date, addressing these questions is needed to transition VCA from research to standard of care and its insurance coverage inclusion (17). It is interesting to note that ethical and psychosocial issues raised by our transplantation field has often led the wider medical field. Our definitions of life and death, and more recently normothermic regional perfusion (NRP)—donor circulatory death (DCD) organ donation challenge existing dogma.

Fifth, the field has started a scientific debate about body-to-head transplantation (BHT), perhaps the final frontier of organ transplantation (18, 19). At first unrealistic, unethical, and futile, but a quixotic unreachable dream for some. The idea would be to save the lives of individuals who suffer from terminal diseases but whose heads and brains are healthy. Obviously, the ethical, surgical, immunological, and psychosocial hurdles associated with human BHT are extraordinarily complex and, at least at present, lack any adequate experimental foundation. We do not know what the future brings, but at one point, we should ask: How many body parts can you exchange and still think of yourself as yourself?

The ever-evolving field of transplantation has never been challenged as it is today by the complexity of problems. By communicating the bench-to-bedside progress across all aspects of organ, tissue, and cell transplants, “Frontiers in Transplantation” provides a much needed global platform for multidisciplinary crosstalk between academia researchers, clinical scientists, and the biotech industry. It is expected that such a concerted effort will remove any remaining boundaries in offering life-saving transplants in the care of patients with terminal organ failure. At the same time, targeting the global epidemic of overweight and obesity—“globesity”—is warranted to prevent serious health disorders, which otherwise require “transplantation” as the only therapeutic option.

## Author Contributions

The author confirms being the sole contributor of this work and has approved it for publication.

## Conflict of Interest

The author declares that the research was conducted in the absence of any commercial or financial relationships that could be construed as a potential conflict of interest.

## Publisher's Note

All claims expressed in this article are solely those of the authors and do not necessarily represent those of their affiliated organizations, or those of the publisher, the editors and the reviewers. Any product that may be evaluated in this article, or claim that may be made by its manufacturer, is not guaranteed or endorsed by the publisher.
